# CD8^+^ T Cell-Induced Expression of Tissue Inhibitor of Metalloproteinses-1 Exacerbated Osteoarthritis

**DOI:** 10.3390/ijms141019951

**Published:** 2013-10-08

**Authors:** Jeng-Long Hsieh, Ai-Li Shiau, Che-Hsin Lee, Shiu-Ju Yang, Bih-O Lee, I-Ming Jou, Chao-Liang Wu, Shun-Hua Chen, Po-Chuan Shen

**Affiliations:** 1 Department of Nursing, Chung Hwa University of Medical Technology, Tainan 717, Taiwan; E-Mail: pipi58871053@yahoo.com.tw; 2 Department of Microbiology and Immunology, College of Medicine, National Cheng Kung University, Tainan 701, Taiwan; E-Mails: alshiau@mail.ncku.edu.tw (A.-L.S.); shunhua@mail.ncku.edu.tw (S.-H.C.); 3 Department of Microbiology, School of Medicine, China Medical University, Taichung 404, Taiwan; E-Mail: chlee@mail.cmu.edu.tw; 4 Institute of Basic Medical Science, College of Medicine, National Cheng Kung University, Tainan 701, Taiwan; E-Mail: shiujuy1016@ntu.edu.tw; 5 Department of Nursing, Chang Gung University of Technology, Puzih, Chiayi County 613, Taiwan; E-Mail: bih-olee@gw.cgust.edu.tw; 6 Department of Orthopedics, College of Medicine, National Cheng Kung University, Tainan 701, Taiwan; E-Mail: jming@mail.ncku.edu.tw; 7 Department of Biochemistry and Molecular Biology, College of Medicine, National Cheng Kung University, Tainan 701, Taiwan; E-Mail: wumolbio@mail.ncku.edu.tw; 8 Department of Orthopedic Surgery, Tainan Hospital, Department of Health, Executive Yuan, Tainan 700, Taiwan

**Keywords:** CD8^+^ T cells, osteoarthritis, TIMP-1, VEGF, MMP-13

## Abstract

Despites the fact that T cells are involved in the pathogenesis of osteoarthritis (OA) little is known about the roles of CD8^+^ T cells in this disease. We investigated the effects of CD8^+^ T cells and the expression of tissue inhibitor of metalloproteinases 1 (TIMP-1) on joint pathology. Using anterior cruciate ligament-transection (ACLT), OA was induced in mice. The knee joints were histologically assessed for manifestations of OA. The CD8^+^ T cells from splenocytes and synovium were flow-cytometrically and immunochemically evaluated, respectively. Local expression of TIMP-1, matrix metalloproteinase (MMP)-13, and VEGF were examined. Cartilage degeneration was slower in CD8^+^ T cell knockout mice than in control mice. CD8^+^ T cells were activated once OA was initiated and expanded during OA progression. More CD8^+^ T cells from splenocytes expressed TIMP-1 in ACLT-group mice than in Sham-group mice. The number of TIMP-1-expressing CD8^+^ T cells in OA mice correlated with the disease severity. TIMP-1 expression in cartilage was co-localized with that of MMP-13 and VEGF. TIMP-1 protein was detected in synovium in which angiogenesis occurred. During the pathogenesis of OA, the expression of TIMP-1, VEGF and MMP-13 accompanying with CD8^+^ T cells activation were increased. Furthermore, inhibiting the expression of TIMP-1 in joints could retard the progression of OA.

## 1. Introduction

Osteoarthritis (OA), a chronic and progressive disorder of the joints, is one of the most prevalent diseases in humans. Factors that cause OA are multiple; they include age, weight, gender, bone density, trauma history, and gene-based susceptibility [1]. Therefore, OA may be best thought of as a group of disorders with varied etiologies whose final common clinical phenotypes converge to create the complex pathophysiology of this disease. Abnormal forces acting on normal cartilage or normal forces acting on abnormal cartilage may cause structural changes in joints: osteophyte formation, synovial inflammation, and cartilage loss.

Angiogenesis, the growth of new blood vessels in both the osteochondral junction and the synovial membrane, is significant in the pathogenesis of OA. By stimulating ossification in the articular cartilage and by transporting monocytes and macrophages in the synovium, angiogenesis promotes osteophyte formation and synovial inflammation [2,3]. The cytokines released from these inflammatory cells may produce metalloproteinases and trigger tissue destruction [4]. Current treatments for mild or moderate OA improve symptoms. Joint replacement surgery is often required in late-stage OA. The efficacy of treatment depends on the severity of the factors involved in the exacerbation of the disease.

Owing to various etiologies, considering OA as a solely degenerative joint disease is a misnomer. OA is not simply a process of wear and tear, but rather, a more complex disease with inflammatory mediators released by cartilage, bone and synovium. These responses could be activated by external stimuli such as injury or internal mechanisms such as aging leading to increased production of inflammatory mediators [5,6]. Once OA is initiated, cartilage fragments fall into the joints. In response to the stimuli, immune cells are recruited into the synovium. Synoviocytes react by producing inflammatory mediators to recruited lymphocytes. Secreted inflammatory molecules, such as interleukin (IL)-1β and tumor necrosis factors (TNFs), in particular, control the degeneration of articular cartilage matrix. Apart from IL-1β and TNF-α, several other cytokines including IL-6, IL-15, IL-17, IL-18, IL-21, leukemia inhibitory factor and IL-8 have been shown to be implicated in OA progression [7]. Chondrocytes produced a variety of matrix-degrading enzymes, including metalloproteinases and aggrecanase. The expressions of these degradative enzymes are major contributors to cartilage degradation.

There is strong evidence that T cells are significantly involved in the inflammation that occurs during the pathogenesis of OA. More than 50% of patients with OA have mononuclear cell infiltrates consisting of CD3^+^ T cells in the synovial membrane [8]. Lymphocyte populations include T lymphocytes expressing CD4^+^, CD8^+^, and CD3^+^/CD4^−^/CD8^−^ T lymphocytes [9,10]. Chondrocytes express major histocompatibility complex (MHC) class II molecules [11]. Peripheral mononuclear cells and T cells react to autologous chondrocytes via HLA, CD4, or CD8 molecules [12]. The infiltration of CD8^+^ and CD4^+^ was associated with early stages of OA [13,14]. In accordance with the previous studies, we found that both the CD8^+^ and CD4^+^ T cells infiltrated the synovium 30 days after OA induction. CD8^+^ and CD4^+^ T cells may act as a primary activator to exacerbate synovitis and/or a secondary regulator to destruct the cartilage. Inflammatory mediators stimulated by T cells would be responsible for the disease manifestations. We previously showed the CD4^+^ T cells promoted the osteoclast formation through the expression of macrophage inflammatory protein-1γ (MIP-1γ) [15]. Nevertheless, the role of infiltrated CD8^+^ T cells in the pathogenesis of OA is unknown. To identify the potential genes affected by CD8^+^ T cells will answer these questions. In the present study, a cytokine array analysis was used to test the genes affected by CD8^+^ T cells in a mouse model of anterior cruciate ligament-transection (ACLT)-induced OA. We found that among the inflammatory mediators we tested, the most affected gene by CD8^+^ T cells was tissue inhibitor of metalloproteinases 1 (*TIMP-1*). The altered expression of TIMP-1 would involve the manifestation of OA.

## 2. Results

### 2.1. OA-Induced Pathological Changes in CD8^−/−^ Mice

**Figure 1:**
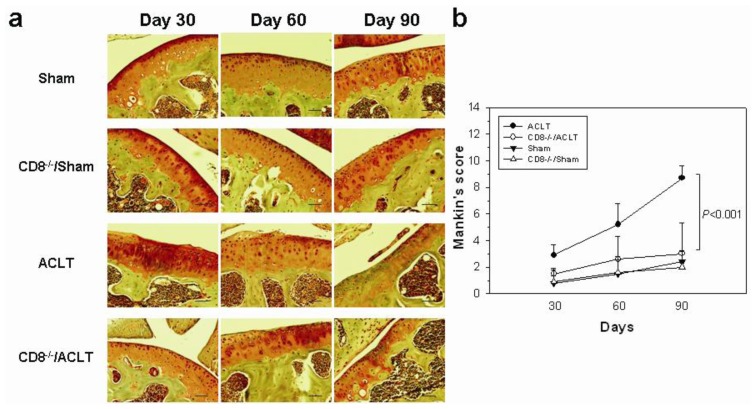

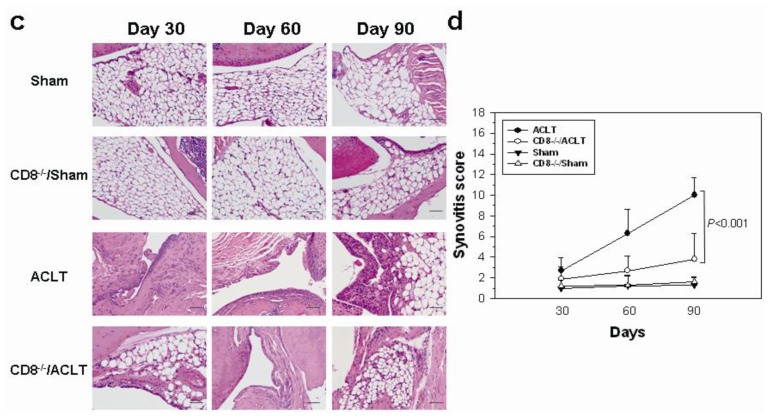
Evaluation of histological changes in the knee joints of anterior cruciate ligament-transection (ACLT)-induced osteoarthritis (OA). The mice were divided into groups by those not subjected to ACLT (Sham-group and CD8^−/−^/Sham group) and those that were subjected to ACLT (ACLT-group and CD8^−/−^/ACLT group). The histological changes at day 30, 60, and 90 after ACLT were analyzed. Cartilages from the medial femoral condyle are shown. (**a**) Representative sections of cartilage which scores were the most close to the means in each group were shown (Safranin-O/fast green stain; 200× magnification; scale bar = 50 μm); (**b**) Histological examination showed a significantly lower Mankin’s score in the joints from CD8^−/−^/ACLT-group mice than in those from ACLT-group mice on day 90; (**c**) Representative sections of synovium which scores were the most close to the means in each group were shown (hematoxylin and eosin stain; 200× magnification; scale bar = 50 μm); and (**d**) Histological examination showed a significantly lower synovitis score in the joints from the CD8^−/−^/ACLT-group mice on day 90. Data are means ± 95% confidence intervals (*n* = 5 per group). Significant difference in scores was observed 90 days after ACLT (*p* < 0.001).

We first examined the effects of CD8^+^ T cells during the progression of OA. Using ACLT, OA was induced in the knee of one hind leg of mice on day 0. The knee cartilage and synovial specimens were removed on day 30, 60, and 90. 30 days after surgery, the irregularity on the superficial layer of cartilage and the proliferation of the lining cells in the synovium was observed in ACLT group mice (Figure 1a,c). The fissure lesions on the surface were noted on day 60, and the disease progressed to a moderate loss of Safranin-O staining and a disappearance of the surface layer cells on day 90. The disease progression in CD8^−/−^/ACLT-group was slower. Specimens from this group showed a slight reduction of Safranin-O staining and irregularity on the surface of the cartilage at day 60 after surgery. A proliferation of chondrocyte in the transitional layer of cartilage was observed on day 90. In the Sham and CD8^−/−^/Sham groups, the superficial cartilaginous layer shows a smooth and regular surface. There is no significant alteration in CD8^−/−^/Sham and Sham groups. The mean osteoarthritic score in the joints of CD8^−/−^/ACLT-group mice was significantly lower than that in the joints of ACLT mice (*p* = 0.0002) (Figure 1b). Synovia in the ACLT group showed hyperplasia and hypertrophy of synovial layer and proliferation of granulation tissue on day 90. Lesions from CD8^−/−^/ACLT-group mice were less severe. The synovial membranes in the CD8^−/−^/ACLT group mice showed more cell proliferation and infiltration than sham-operated mice when disease progressed. The structure of cartilage and synovium in both of the sham-operated groups (Sham-group and CD8^−/−^/Sham group) appeared normal. The mean synovitis score in the joints of CD8^−/−^/ACLT-group mice was significantly lower than that in the joints of ACLT mice 90 days after OA induction (*p* = 0.0004) (Figure 1d). The intra class coefficients of both scores used for evaluating interobserver’s variation at day 30, 60, and 90 were 0.64, 0.88, and 0.97, respectively, *p* < 0.001.

### 2.2. CD8^+^ T Cell Activation during the Progression of OA

**Figure 2:**
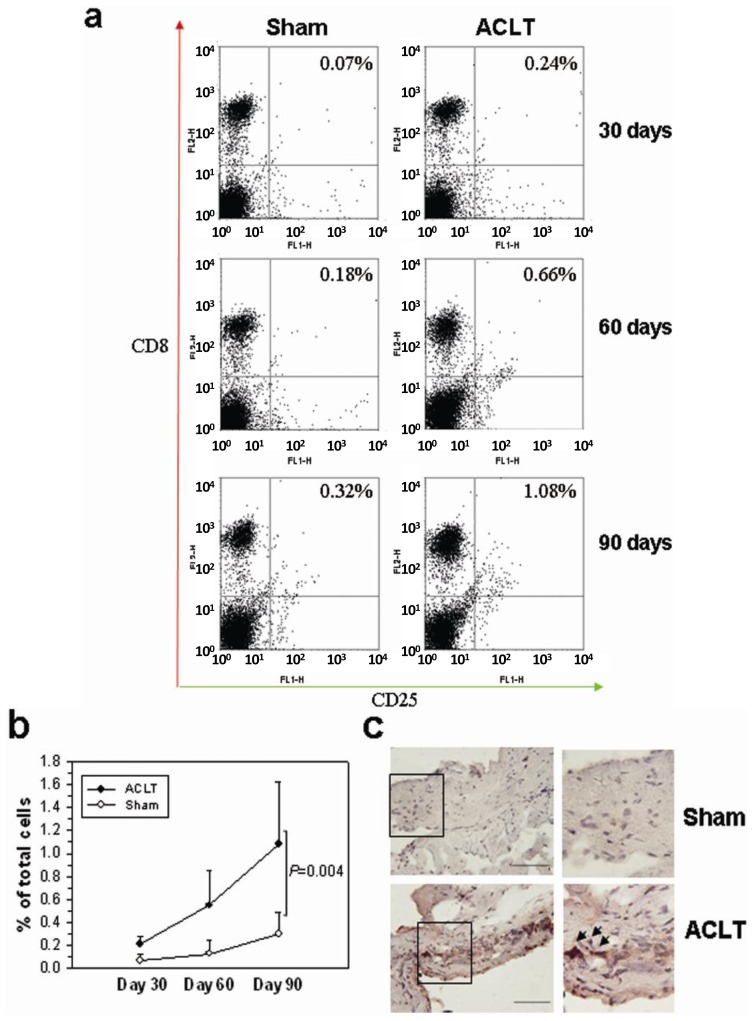
Quantitation of CD8^+^ T cells in mice with OA. (**a**) Splenocytes of four mice per group were stained for surface CD8 and CD25 on day 30, 60 and 90. Data are expressed as the percentage of CD8^+^/CD25^+^ T cells/1 × 10^6^ splenocytes. Representative flow cytometry data from each group are shown; (**b**) The number of activated CD8^+^ T cells in the ACLT group increased remarkably during the progression of OA. Data are as means ± 95% confidence intervals. Significant difference was showed 90 days after ACLT (*p* = 0.004); and (**c**) Synovial membranes from Sham- and ACLT-group mice on day 90 were removed and stained with antibody against CD8. The infiltrated CD8^+^ T cells were indicated with arrows. Representative sections of synovium in each group are shown [Immunohistochemistry (IHC) stain; 200× magnification; scale bar = 200 μm; the inset represents the magnified area; ×400 magnification].

We next tested if CD8^+^ T cells could be activated when OA was induced. Flow-cytometry was used to count the number of activated CD8^+^ T cells in the splenocytes of the Sham and ACLT groups on day 30, 60 and 90. The percentage of CD8^+^/CD25^+^ T cells in the ACLT group was higher on day 30, 60 and 90 after OA induction (Figure 2a). In the representative data, 90 days after ACLT, the activated CD8^+^ T cells in ACLT group were more than three times as those in Sham group. The percentage of activated CD8^+^ T cells was significantly higher in the ACLT group than in the Sham group on day 90 [1.08% (0.54–1.62) *vs.* 0.32% (0.11–0.49); *p* = 0.004] (Figure 2b). Furthermore, there was notable infiltration of CD8^+^ T cells into the synovium of ACLT-group mice on day 90 (Figure 2c, arrows), but there was no significant change in the Sham-group mice. These data suggest that the CD8^+^ T cell in mice can be activated from disease initiation to subsequent progression. This activation may be responsible for exacerbation of the disease.

### 2.3. Decreased TIMP-1 Expression in CD8^−/−^ Mice

**Figure 3:**
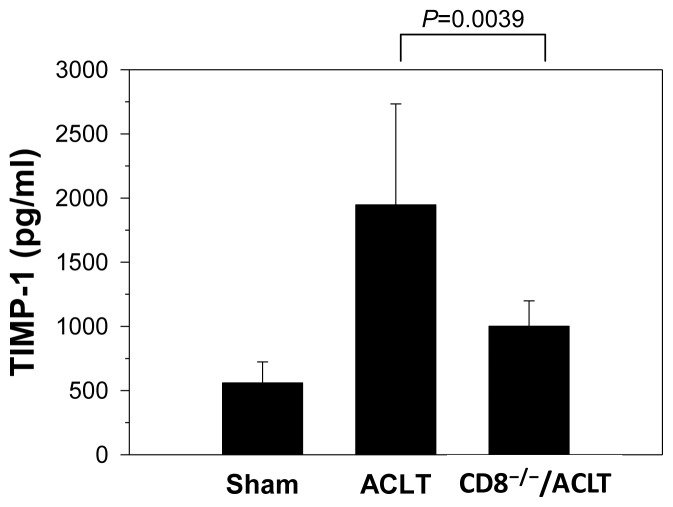
The levels of TIMP-1 in joints after OA induction. TIMP-1 expression was significantly higher in ACLT-group mice than in Sham- and CD8^−/−^/ACLT-group mice (determined using ELISA). Values are means ± 95% confidence intervals (*n* = 7 per group). Results are representative of three independent experiments.

To identify the proteins regulated by CD8^+^ T cells in joints, we induced OA in CD8^−/−^ mice and then performed a cytokine array. On day 90 at mice sacrifice, the synovial tissues were removed and dissected for homogenization. The homogenates from five mice in each group were pooled. TIMP-1 expression in mice after ACLT was determined using a mouse inflammation antibody array kit. The array analysis showed that three cytokines and chemokines—soluble tumor necrosis factor receptors II (sTNF-RII), IL-4, and tissue inhibitor of metalloproteinase (TIMP)-1—were top-regulated in the CD8^−/−^/ACLT-group mice on day 90 after OA induction. The three proteins are shown in Table 1, with their respective fold-change. The expression of sTNF-RII and IL-4 was lower, but the expression of TIMP-1 was higher in the ACLT group than in the CD8^−/−^/ACLT group. This result was confirmed using an enzyme-linked immunosorbent assay (ELISA). TIMP-1 expression was significantly lower in the CD8^−/−^/ACLT group [1001.53 (804.83–1198.23) pg/mL] than in the ACLT group [1947.12 (1160.47–2733.77) pg/mL; *p* = 0.0039] (Figure 3).

### 2.4. Increased TIMP-1 Expression in the CD8^+^ T Cells after OA Induction

**Figure 4:**
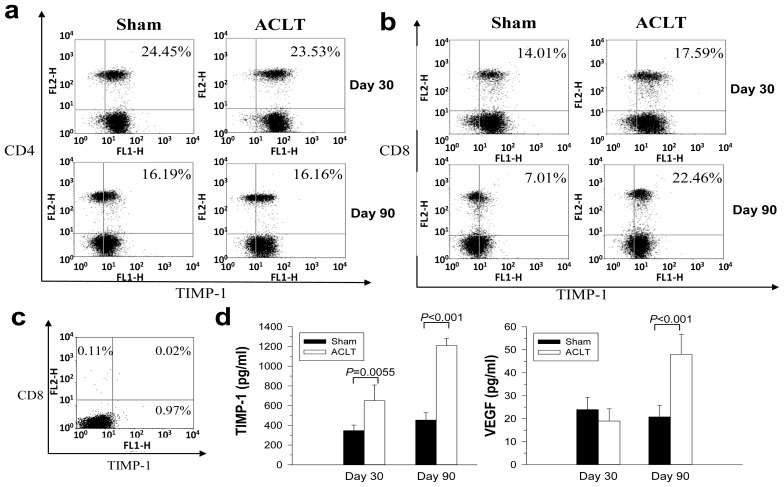
TIMP-1-expressing T cells in spleens and tissue after OA induction. Intracellular flow cytometry of TIMP-1 in splenocytes 30 days and 90 days after OA induction (*n* = 6 per group). The cells were double-stained with (**a**) CD4 and TIMP-1 markers; or (**b**) CD8 and TIMP-1 markers. Representative data in each group are shown; (**c**) Cells stained with secondary antibodies only. Results are representative of two independent experiments; and (**d**) Levels of TIMP-1 and VEGF in joint extracts from the ACLT and Sham groups were determined using ELISA. Values are means ± 95% confidence intervals. Results are representative of two to three independent experiments.

**Figure 5:**
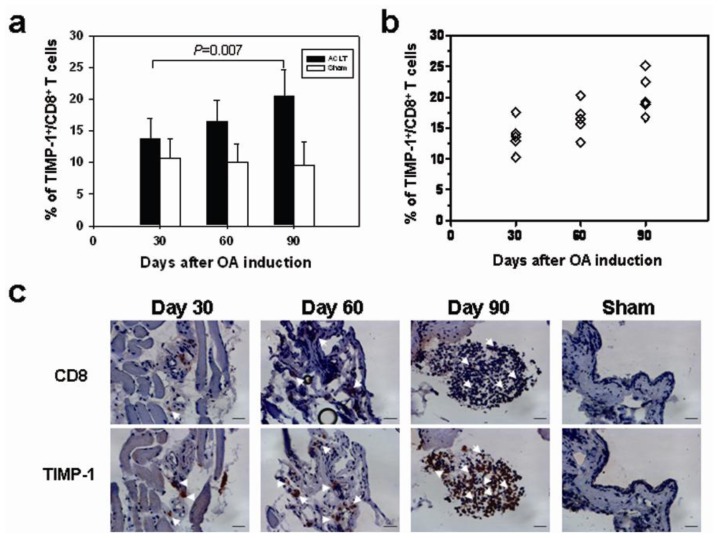
Increase of TIMP-1-expressing CD8^+^ T cells along with OA progression. (**a**) The percentage of TIMP-1-expressing CD8^+^ T cells in spleens was determined by flow cytometry (*n* = 5 in each group). Data are means ± 95% confidence intervals. The disease severity was classified by three time points (30, 60 and 90 days) after OA induction; (**b**) Spearman’s correlation was used to determine the correlation between the percentage of TIMP-1-expressing CD8^+^ T cells, and the severity of OA, *p* = 0.0011; and (**c**) Synovial membrane from the Sham and ACLT groups on day 30, 60 and 90 were removed and stained with antibodies against CD8^+^ and TIMP-1. The Sham group on day 90 was shown. In the ACLT group mice, increased TIMP-1-expressing CD8^+^ T cell infiltration was seen in joints compared to that in the Sham group 90 days post-surgery (400× magnification; scale bar = 20 μm). The CD8- and TIMP-1-double positive cells were indicted with white arrows. The TIMP-1-positive cells were indicated with arrow heads.

Among tested cytokines, TIMP-1 was the most reduced in CD8^−/−^ mice. We therefore examined the expression of TIMP-1 from T cell population of OA mice. Ninety days after OA induction, cells were analyzed for T cell surface markers and intracellular expression of TIMP-1 from the splenocytes of the mice. A certain proportion of CD4^+^ T cells in the spleens constitutively expressed TIMP-1 on day 30 and 90; no increase of TIMP-1 expression was seen following OA induction (Figure 4a). The complete data were shown in Supplementary Table S1. The number of CD8^+^ T cells expressing TIMP-1 in the ACLT group on day 30 was higher than that in the Sham group (17.59% *vs.* 14.01%), which increased more remarkably on day 90 (22.46% *vs.* 7.01%) (Figure 4b). The background obtained from intracellular staining of TIMP-1 was shown in Figure 4c. Furthermore, TIMP-1 expression from the joints in the ACLT group was significantly higher than that in the Sham group on day 30 [650.5 (491.6–809.4) pg/mL *vs.* 346.74 (290.2–403.28) pg/mL; *p* = 0.0055], and its increase was seen more significantly in the ACLT group on day 90 [1209.87 (1138.39–1281.35) pg/mL *vs.* 453.52 (378.63–528.41) pg/mL; *p* < 0.001] (Figure 4d). VEGF expression was not significantly different on day 30, but it was significantly higher in ACLT-group mice than in Sham-group mice on day 90 [47.92 (39.13–56.71) pg/mL *vs.* 20.8 (15.74–26.4) pg/mL; *p* < 0.001] (Figure 4d). Increased TIMP-1 expression was observed in both of the CD8^+^ T cells and joint tissues. These results suggested that CD8^+^ T cells may produce the TIMP-1 themselves or induce other cells, such as chondrocytes in joints to produce TIMP-1. We further tested if the increased production of TIMP-1 correlated to the disease severity using flow cytometry. The number of TIMP-1-expressing CD8^+^ T cells in the spleens increased with the disease progression (Figure S1). Compared with mice 30 days after ACLT [13.70% (10.2%–17.6%)], the percentage of CD8^+^ T cells expressing TIMP-1 in spleens was significantly increased in mice 90 days after ACLT [20.5% (16.4%–24.67%); *p* = 0.007] (Figure 5a). The association between the percentage of CD8^+^ T cells expressing TIMP-1 and disease severity was further confirmed. The number of TIMP-1-expressing CD8^+^ T cells in spleens correlated with disease severity (Spearman’s *p* = 0.6992, 95%CI 0.2758–0.8954, *p* = 0.0037) (Figure 5b). On day 90 after ACLT, there was no CD8^+^ T cells detected in joints of Sham group mice whereas many CD8^+^ T cells infiltrated throughout the synovial membranes of the ACLT group mice. The number of TIMP-1-expressing CD8^+^ T cells and other TIMP-1-expressing cells in synovial membranes increased when disease progressed (Figure 5c). These results indicated that an increased number of TIMP-1-expressing CD8^+^ T cells both in spleens and synovial tissues of mice correlated to the disease severity. Through direct secretion of TIMP-1, the activated CD8^+^ T cells may affect the OA disease progression.

### 2.5. Evaluation of TIMP-1, VEGF, and MMP-13 Expression in Joints with ACLT-Induced OA

**Figure 6:**
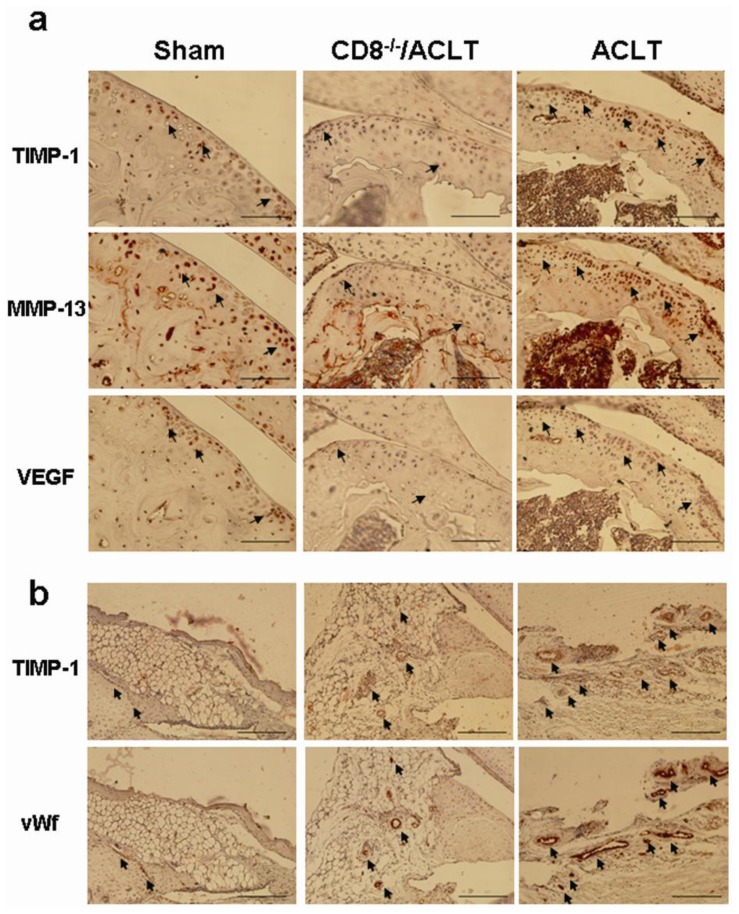
Immunohistochemical staining for TIMP-1, MMP-13, and VEGF in the joints of mice with OA. Representative immunohistochemical images of the cartilage and synovium are shown (*n* = 5 per group) (**a**) Higher TIMP-1, MMP-13, and VEGF expression was seen in the superficial cartilage and in the osteochondral cartilage junctions of ACLT-group mice than in Sham- and CD8^−/−^/ACLT-group mice 90 days post-surgery (IHC stain; 200× magnification; scale bar = 50 μm). The overlapped locations of three gene expressions are indicated with arrows; and (**b**) Higher TIMP-1 expression and more abundant angiogenesis were seen in ACLT-group mice synovia than in Sham- and CD8^−/−^/ACLT-group mice synovia (IHC stain; 200× magnification; scale bar = 50 μm). The overlapped locations of TIMP-1 and vWf expression are indicated with arrows.

TIMP-1 expression induces MMP-13 expression, which is a key mediator of cartilage degradation in OA. We next examined the MMP-13 expression by IHC in cartilage. Interestingly, the expression of MMP-13 was co-localized with the TIMP-1 expression in the superficial and osteochondral area of cartilage in ACLT-group mice 90 days post-surgery (Figure 6a). The expression of both MMP-13 and TIMP-1 was significantly lower in the CD8^−/−^/ACLT-group mice than in ACLT-group mice. In addition, VEGF expression was co-localized with TIMP-1 expression in the same areas. Co-localization of TIMP-1 expression and angiogenesis also occurred in the synovia (Figure 6b). TIMP-1 expression was much more abundant in the subsynovial membrane of ACLT-group mice than in that of CD8^−/−^/ACLT- and Sham-group mice.

### 2.6. *In Vivo* Histopathologic Evaluation in Mice with OA after Treatment with Neutralization TIMP-1 Antibody

**Figure 7:**
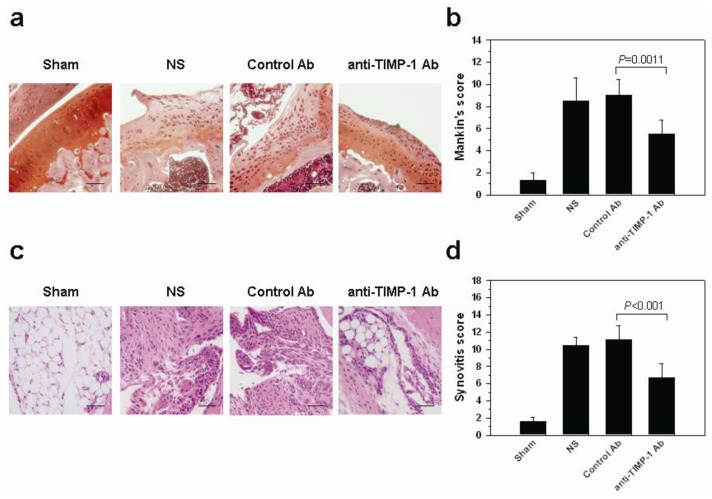
Histological analysis in the knee joints of TIMP-1 neutralization antibody-treated mice (*n* = 5 per group). Cartilages from the medial femoral condyle are shown. (**a**) Representative sections of cartilage whose scores were closest to the mean in each group were shown (Safranin-O/fast green stain; 200× magnification, scale bar = 50 μm). The specimens from mice treated with NS and control antibody showed remarkably decreased Safranin-O staining and surface irregularities. The TIMP-1 antibody-treated specimens had an irregular superficial layer of cartilage. The surface layer of cartilage in the Sham group was smooth and showed no significant changes; (**b**) Histologic examination showed a reduced Mankin’s score in the medial femoral condyles of cartilage from mice treated with TIMP-1 antibody. Each value represents the means ± 95% confidence intervals. Significant difference in scores was observed between control Ab and anti-TIMP-1 Ab groups (*p* = 0.0011); (**c**) Representative sections of synovium which scores were the most close to the means in each group were shown (hematoxylin and eosin stain; 200× magnification, scale bar = 50 μm). Synovia in the NS- and control antibody-treated mice showed hyperplasia and hypertrophy of synovial lining cells. The TIMP-1 antibody-treated specimens showed slightly more cell proliferation in the synovial lining and cell infiltration than did the sham-operated specimens; and (**d**) Histologic examination showed a reduced synovitis score in the synovial tissues of knees from mice treated with TIMP-1 antibody. Each value represents the means ± 95% confidence intervals. Significant difference in scores was observed between control Ab and anti-TIMP-1 Ab groups (*p* = 0.003).

To investigate the role of TIMP-1 in the OA, we inhibited the local expression of TIMP-1 when OA was induced. One week after surgery, TIMP-1 neutralization antibody was intraarticularly injected in the OA knees of mice (0.5 mg/kg) once a week for two consecutive weeks. 30 days at sacrifice, cartilages from the mice treated with either NS (normal saline) or an isotype-matched goat IgG antibody (control Ab) showed a remarkably reduction of Safranio-O staining and loss of chondrocytes of surface layer cells (Figure 7a). Nevertheless, in the joints treated with anti-TIMP-1 antibody (anti-TIMP-1 Ab), the severity of lesion was reduced. Slight reduction of Safranin-O staining and some chondrocytes cloning were observed on the superficial layer of cartilage. In the sham-operated knee joints, most of the articular cartilage of the femur had a smooth surface, evenly stained with Safranin-O. The Mankin’s score in the anti-TIMP-1 Ab-treated joints was significantly lower than that in the control Ab-treated ones [5.5 (4.18–6.82) *vs.* 9.0 (7.54–10.46), *p* = 0.0011, Figure 7b]. Synovia in NS and control Ab treated-group mice showed hyperplasia and hypertrophy of the lining cells and increased infiltration of inflammatory cells. As compared with the sham-operated group, a slight increase of synovial lining cell proliferation and inflammatory cell infiltration was noted in the anti-TIMP-1 Ab-treated group mice (Figure 7c). The synovitis score in joints treated with anti-TIMP-1 Ab was significantly lower than that treated with control Ab [6.7 (5.02–8.38) *vs.* 11.1 (8.72–13.48), *p* = 0.003, Figure 7d]. The intra class coefficients of both scores used for evaluating interobserver’s variation was 0.82, *p* < 0.001. Taken together, these results indicated that the expression of TIMP-1 increased in the cartilage and synovium of OA mice. Inhibiting its expression could retard the disease progression and attenuate the severity of OA.

## 3. Discussion

To determine the effect of CD8^+^ T cells on OA development, we induced OA in the knee joints of CD8^−/−^ mice (CD8^−/−^/ACLT). We found that cartilage degeneration was slower in CD8^−/−^ mice than in wild-type mice after ACLT. The levels of three proteins in the knee joints of CD8^−/−^/ACLT mice had changed: sTNF-RII and IL-4 had increased, and TIMP-1 had decreased. sTNF-RII is a natural inhibitor of TNF-α. IL-4 is a potent inhibitor of IL-1β because of its ability to induce production of the IL-1 receptor antagonist [16]. They are both believed to have anti-inflammatory functions during disease progression [17,18]. The OA symptoms may be partially improved through the reversion of these anti-inflammatory factors when CD8^+^ T cells are absent. The cytokine array assay showed that the largest change in protein level was in TIMP-1. An ELISA assay showed a 48.6% reduction in TIMP-1 expression from the joint homogenate of CD8^−/−^/ACLT mice when OA was induced. TIMP-1 is one member of the TIMP family and a dominant TIMP, which inhibits all collagenases, including MMP-13 [19,20]. TIMP-1 was thought to principally regulate extracellular matrix (ECM) turnover through modulating the degradative activity of MMPs. The disturbance of the balance between TIMP-1 and MMPs may result in an excess of MMPs over TIMP-1, which underlies pathologic cartilage destruction. Our previous data also showed that there was a mild increase of TIMP-1 in CD4^−/−^/ACLT mice compared to ACLT mice on day 90 post-surgery [15] implying an opposite effect of CD4^+^ T cells in the regulation of TIMP-1. Cytokines produced by Th1 cells could decrease the production of TIMP-1 [21]. Since neither the number of activated CD4^+^ T cell nor the percentage of TIMP-1-expressing CD4^+^ T cells were significantly changed between ACLT- and Sham-group mice 90 days after surgery, the effect of CD4^+^ T cells on the TIMP-1 expression may be limited at later stages of OA.

We showed that the CD8^+^ T cells in mice could be activated from the initiation of OA (day 30) to the subsequent disease progression (day 90). During the differentiation of T cells, large numbers of CD8^+^CD25^+^ T cells appear in the thymus, and then they disappear after entering the spleen. The CD25 is an α chain of the IL-2 receptor that enhances the sensitivity of lymphocytes to IL-2, which could be referred as an activation marker of T cells in spleens. A minor but significant fraction of CD8^+^CD25^+^ T cells could be detected in the spleens when CD8^+^ T cells were activated [22]. Our results showed that the percentage of CD8^+^CD25^+^ T cells in spleens were both increased in ACLT groups 60 and 90 days after OA induction. Some of them expressed low level of CD8^+^ markers (CD8dim). Therefore, after ACLT, in addition to an increase in the total number CD8^+^ T cells, a specific subpopulation of TIMP-1 expressing CD8dim (T cytotoxic subset) may be enriched. The percentage of TIMP-1-expressing CD8^+^ T cells was higher in ACLT-group mice than in Sham-group mice, both 30 days and, especially, 90 days after ACLT. The number of CD8^+^ T cells expressing TIMP-1 in the ACLT group on day 90 was remarkably higher than that in the Sham group. There were no significant changes in the percentage of TIMP-1-expressing CD4^+^ T cells, however. We also showed that the number of TIMP-1-expressing CD8^+^ T cells in spleens was increased with the disease progression of OA (Figure 5a). The number of CD8^+^ T- and TIMP-1-double positive cells in spleens correlated with the disease severity. Moreover, the number of TIMP-1-expressing CD8^+^ T cells and TIMP-1-expressing choncrocytes and synoviocytes in joints increased when the disease progressed. These results suggested that the activated CD8^+^ T cells affect the OA disease progression either through direct release of TIMP-1 or stimulate other cells to express TIMP-1 in joints. Because a significant increase in TIMP-1 was seen 30 days after ACLT—earlier than an increase in VEGF (90 days after ACLT, Figure 4d)—the expression of TIMP-1 and VEGF in OA joints may have correlation. How the CD8^+^ T cells regulate the VEGF required further investigation.

TIMP-1 produced by CD8^+^ T cells inhibited parasite clearance during chronic *Toxoplasma* infection in the brain [23]. The infiltrating CD8^+^ T cells were able to initiate VEGF expression to promote vascular permeability of the central nervous system under neuroinflammatory conditions [24]. It has been suggested [25] that the interaction of chondrocytes with autologous T cells increased the production of MMP1, MMP3, and MMP-13. Osteochondral and synovial angiogenesis is pivotal in the pathogenesis of OA and is highly associated with the severity of OA symptoms and synovitis [3]. Both *VEGF* and *TIMP-1* are upregulated in patients with OA, and higher scores are associated with greater chondropathy [26]. Consistent with prior studies, we found that VEGF and TIMP-1 in the joints in OA samples from mice had both increased. We also found a higher density of blood vessels in the synovia of OA-group mice. The expression of TIMP-1, MMP-13, and VEGF were in the same locations in tissue, which suggested that these factors are likely to be secreted from the same areas, and even the same cells (such as chondrocytes in cartilage, or infiltrated monocytes and lymphocytes in synovium) when OA is induced. VEGF induced the secretion of MMP-1, MMP-3, and, especially, MMP-13 in an immortalized chondrocyte cell line [27]. Furthermore, the MMP-13 levels were highly associated with the VEGF levels in the synovial fluid of OA patients [28]. The coordinated upregulation of these genes indicated that *TIMP-1* may contribute to OA severity both as a regulator of ECM turnover and of angiogenesis. The CD8^+^ T cells may modulate angiogenesis and cartilage destruction through the persistent expression of *TIMP-1* during the progression of OA.

Although *TIMP-1* was thought to principally regulate MMPs activity. The relation between these genes *in vivo* may be more complicated than thought. It is now recognized that *TIMPs*, like *MMPs*, are pleiotropic and affect cell growth, migration, and apoptosis. *TIMP-1* regulates cell survival MMP-independently by recognizing specific cell surface receptors [29]. Therefore, the net effects of TIMP-1 depend upon the cellular context involved and the details of the specific model system used [30]. Whether TIMP-1 acts as a protective or a destructive role has not yet been clarified and awaits further investigation. It may depend upon the composition of extracellular matrix proteins within the microenvironments. In normal cartilage and synovium, certain amounts of TIMP-1 and MMPs would be maintained to keep the homeostasis of extracellular matrix. In OA cartilage, infiltrated CD8^+^ T cells, either themselves or by activating chondrocytes, secrete the inflammatory mediator TIMP-1 to the cartilage. TIMP-1 here may act as a matrix regulator to balance the increased MMPs expression. Our data showed that when OA was initiated, both of the TIMP-1 and MMP-13 were increased in the superficial cartilage (Figure 6). In OA synovium, CD8^+^ T cells may also exert as a direct source of TIMP-1 and stimulate synoviocytes to express TIMP-1. TIMP-1 here may act as an angiogenesis activator to allow vessel formation. Our data showed that both TIMP-1 expression and vessel formation was co-localized in the synovium. Changes of TIMP-1 expression in cartilage and synovium may be spatially correlated, indicating that they both contributed to the disease progression. The net effect of increased TIMP-1 and MMP-13 expression lead to loss of homeostasis in joints and resulted in cartilage damage.

The animal model we used in Figure 1 is the ACLT model. The reason we chose this type of model is that the disruption of the anterior cruciate ligament (ACL) is common in older adults with knee OA. In a magnetic resonance imaging study, approximately 23% of people with symptomatic knee OA had evidence of complete ACL rupture, but less than half of those gave a history of trauma [31]. Following acute ACL injury, biomarkers of inflammation and collagen loss can be detected at higher levels in synovial fluid of the affected knee [32]. It indicated that synovitis reflected preclinical disease during early posttraumatic phase, which could impact the subsequent progression of the disease. However, this model has disadvantages. The ACLT caused the joint instability and its effect was classified as a secondary OA. The etiology is different from that of human primary OA. Therefore, the involvement of CD8^+^ T cells and inflammatory components of primary OA may not be the same as observed in mice ACLT model. Furthermore, the ACLT model gave severe OA and may involve subchondral bone erosion. The animal model where we induced OA in Figure 7 was ACLT in combination with medial menisci (MM) removal (ACLT/MM). A moderate to severe OA in mice could be achieved by the later one. Nevertheless, compared to ACLT and ACLT/MM models, destabilization of the medial meniscus (DMM) instability surgical model is more consistent with lesions observed in aged spontaneous mouse models of OA [33]. It would probably be more suitable for analyzing the effect of gene deletions on potential targets in OA.

## 4. Experimental Section

### 4.1. Animal Model

Seven-week-old male B6 mice were purchased from the Laboratory Animal Center of National Cheng Kung University and CD8^+^ T cell knockout (CD8^−/−^) mice were purchased from the Jackson Laboratory (Bar Harbor, ME, USA), mouse strain name: B6.129S2-*Cd8a^tm1Mak^*/J. The experimental protocol adhered to the rules of the Animal Protection Act of Taiwan and was approved by the university’s Laboratory Animal Care and Use Committee. The mice were divided into groups that were not subjected to anterior cruciate ligament-transection (ACLT) (Sham-group and CD8^−/−^/Sham group) and that were subjected to ACLT (ACLT-group and CD8^−/−^/ACLT group). The mice in ACLT-group and Sham-group were wild-type mice. The mice in CD8^−/−^/ACLT and CD8^−/−^/Sham group were CD8^+^ T cell knockout mice. Both of the experimental-induced OA mice was anesthetized with tiletamine hydrochloride and zolazepam hydrochloride (10 mg/kg) (Zoletil 50; Virbac, Carros, France) and then subjected to modified procedures [34]. In the other independent experimental OA, simultaneous anterior cruciate ligament-transection (ACLT) and medial menisci (MM) removal procedure (ACLT/MM) was performed to induce more severe OA, whereby cartilage damage and synovitis occurred 14 days after surgery [34]. Mice were intraarticularly injected with either goat polyclonal TIMP-1 neutralization antibody (0.5 mg/kg, R&D Systems, Minneapolis, MN, USA) or equivalent doses of an isotype-matched goat IgG antibody (R&D Systems) twice on day 7 and day 14 after surgery. Thirty days later, the knees were removed for histological assessment.

### 4.2. Histology Assessment

To evaluate the histopathologic changes in the cartilage of CD8^+^ T cell knockout mice, ACLT was used to induce OA (ACLT and CD8^−/−^/ACLT) (*n* = 5 per group). After 30, 60 and 90 days, the mice were euthanized and their synovial membranes were removed, fixed, and embedded in paraffin. Serial sections (5 μm thick) were cut and stained with hematoxylin and eosin. The cartilage was processed for staining with Safranin-O/fast green and hematoxylin and eosin. Histological changes in cartilage were scored using Mankin’s histological grading method [35,36], and those in synovial surface tissue and subsynovial tissue were evaluated and scored [35,37]. Briefly, the grading system assigns separate scores based on two categories. (a) Three subcategories of the synovial lining layer: (i) hyperplasia of the synovial lining cells (0–3 points), (ii) hypertrophy of the synovial lining layer (0–3 points), and (iii) the infiltration of inflammatory cells (0–3 points); and (b) Three subcategories of the subsynovial tissue: (i) the proliferation of granulation tissue (0–3 points), (ii) vascularization (0–3 points), and (iii) the infiltration of inflammatory cells (0–3 points). Total scores in each category were calculated; the maximum score was 18 points. All specimens were graded by two blinded musculoskeletal researchers (I.-M.J. and P.-C.S.).

### 4.3. Flow Cytometry

Splenocytes from the Sham and ACLT groups (*n* = 4 per group) were collected on day 30, 60 and 90 after ACLT. Splenocytes (1 × 10^6^ per analysis) were double-stained with PE-conjugated-anti-CD8 (Ly-2; BD Biosciences, San Jose, CA, USA) and FITC-conjugated-anti-CD25 (IL-2 Receptor α chain, p55; BD Biosciences). For detecting TIMP-1-expressing T cells in spleen, splenocytes from the Sham and ACLT groups (*n* = 6 per group) were collected on day 30 and 90 after ACLT. Splenocytes (1 × 10^6^ per analysis) were first stained with either PE-conjugated-anti-CD4 antibody or PE-conjugated-anti-CD8 antibody. The cells were then fixed and permeabilized using a PharMingen’s Cytofix/Cytoperm™ kit (BD Biosciences). The thoroughly permeabilized cells were then stained with TIMP-1 polyclonal antibody (Abcam, Cambridge, UK) which was sequentially incubated with DyLight^®^ 488 conjugated goat anti-rabbit polyclonal antibody (Abcam, Cambridge, UK) for 1 h at 4 °C for flow cytometric analysis. For secondary antibody only, the cells were double stained with PE-conjugated rat IgG2a (eBioscience, San Diego, CA, USA) and Dylight^®^ 488-conjugated goat anti-rabbit IgG (Jackson, Bar Harbor, ME, USA). In the other independent experiment, splenocytes from the Sham and ACLT groups (*n* = 5 per group) were collected on day 30, 60 and 90 after ACLT. Splenocytes (1 × 10^6^ per analysis) were double-stained with PE-conjugated-anti-CD8 antibody and TIMP-1 polyclonal antibody. The cells were then washed twice with staining buffer and suspended in RPMI-based buffer for flow cytometric analysis.

### 4.4. Cytokine Array

On day 90, the mice were euthanized, their joints skinned, and the synovial tissue removed and dissected for homogenization in phosphate-buffered saline (PBS) containing a protease inhibitor cocktail. The homogenates from five mice in each group were pooled. TIMP-1 expression in mice after ACLT was determined using a mouse inflammation antibody array kit (RayBiotech, Atlanta, GA, USA). The intensity of the signals was shown using a digital imaging analysis system (Eastman Kodak, Rochester, NY, USA). The relative density was normalized with an internal control and expressed as a ratio of the image density of each group divided by that of the Sham group. Each value represents the average of two replicated spots on the membrane.

### 4.5. Enzyme-Linked Immunosorbant Assay

Mouse TIMP-1 and VEGF levels in the homogenates (*n* = 6 per group) were quantified using an enzyme-linked immunosorbent assay (ELISA) kit (R&D Systems, Minneapolis, MN, USA).

### 4.6. Immunohistochemical Staining (IHC)

To analyze CD8^+^ T cell infiltrates in the synovium, ACLT was used to induce OA in wild-type mice (*n* = 5), which were euthanized 90 days later. Their synovial membranes were removed under a dissecting microscope and then snap frozen. Cryostat sections (5 μm thick) were cut and then incubated with rat anti-mouse CD8 antibody (1:1000; 53–6.7) (GeneTex, Irvine, CA, USA) at 4 °C overnight. To detect TIMP-1-expressing-CD8^+^ T cells, the synovial membranes and spleens were removed on day 30, 60 and 90 after ACLT and stained with antibodies against CD8^+^ and rabbit anti-mouse TIMP-1 (1:50) (Santa Cruz Biotechnology, Santa Cruz, CA, USA). To test Matrix metalloproteinase (MMP)-13, and VEGF expression, the samples from joints were prepared from wild-type and CD8^−/−^ mice (*n* = 5 per group). Serial sections of cartilage were stained with, MMP-13 (1:50) antibodies (Santa Cruz Biotechnology, Santa Cruz, CA, USA), and goat anti-mouse VEGF (1:50) (Santa Cruz Biotechnology, Santa Cruz, CA, USA) at 4 °C overnight. After they had been sequentially incubated with the appropriate secondary antibody (1:400) (Jackson ImmunoResearch Laboratories, West Grove, PA, USA) for 2 h at room temperature with aminoethyl carbazole as the substrate chromogen (Invitrogen Zymed Laboratories, Camarillo, CA, USA), the slides were counterstained with hematoxylin and eosin. Serial sections of synovium were stained with rabbit anti-mouse TIMP-1 (1:50) (Santa Cruz Biotechnology, Santa Cruz, CA, USA) and factor VIII (von Willebrand’s factor; Dako, Carpinteria, CA, USA). Angiogenesis was visualized using factor VIII-positive vessels.

### 4.7. Statistical Analysis

All data are means plus 95% confidence intervals (CI). JMP 8.0 (SAS Institute Inc., Cary, NC, USA) was used to analyze the histological data in Figures 1b,d and 7b,d. Significance between groups was estimated using one-way analysis of variance (ANOVA). To evaluate the differences between groups, we used Tukey’s Honestly Significant Difference test set at *p* < 0.05. The intra class coefficient (SPSS 15.0; SPSS Inc., Chicago, IL, USA) was used to analyze the interobserver’s variation. The quantification of TIMP-1 and VEGF proteins in Figures 3 and 4b,d were analyzed using Student’s *t*-test. Significance was set at *p* < 0.05. Associations between variables are expressed as Spearman’s correlation coefficients using Prism 5.0 software (GraphPad, San Diego, CA, USA). Significance was set at *p* < 0.05.

## 5. Conclusions

We showed that CD8^+^ T cells were activated once OA was initiated and constitutively proliferated during the progression of OA in a mouse ACLT model. Cytokine array analysis showed that TIMP-1 protein expression was significantly lower when CD8^+^ T cells were ablated. The expression of TIMP-1 by CD8^+^ T cells and other cells in joints was associated with disease severity. The up-regulation of *TIMP-1* could be involved in modulating joint angiogenesis and matrix turnover in OA. Targeting the TIMP-1 expression in joints could attenuate the OA progression. These data provide the potential role of CD8^+^ T cells in the pathogenesis of OA disease. Our findings may provide more implications for new targets for disease therapies.

## Supplementary Information



## Figures and Tables

**Table 1:** Cytokines and cytokine receptor identified by cytokine array analysis.

Gene	Fold-change *	

	ACLT	CD8^−/−^/ACLT
*TIMP-1*	1.90	0.75
*sTNFRII*	0.59	0.86
*IL-4*	0.76	1.06

* The fold-change was expressed as a ratio of the expression level of cytokines and cytokine receptor in ACLT and CD8^−/−^/ACLT groups divided by the expression level in Sham group.
