# Supplementary material for: CD8+ T Cell-Induced Expression of Tissue Inhibitor of Metalloproteinses-1 Exacerbated Osteoarthritis
Source: Int J Mol Sci. 2013 Oct 8;14(10):19951–70. doi: 10.3390/ijms141019951 (PMC3821596; doi:10.3390/ijms141019951)
Supplement: Supplementary file 1 [file ijms-14-19951-s001.pdf]

Supplementary Information

Table S1. The percentage of CD4$^+$/TIMP-1$^+$ and CD8$^+$/TIMP-1$^+$ cells in spleens after osteoarthritis (OA) induction.

**Cells**	**CD4$^+$/TIMP-1$^+$**		**CD8$^+$/TIMP-1$^+$**	
**Group**	**Sham**	**ACLT**	**Sham**	**ACLT**
**Day 30**	24.93 (21.45–28.41)	22.65 (20.98–24.32)	12.57 (11.27–13.87)	15.07 (13.54–16.60) [a]
**Day 90**	16.82 (13.11–20.53)	15.14 (11.05–19.23)	8.30 (5.82–10.78)	21.82 (16.06–27.58) [b]

Sham, the mice were not subjected to anterior cruciate ligament-transection (ACLT); ACLT, the mice were subjected to ACLT to induce OA; Data are means ± 95% confidence intervals and analyzed using Student's *t*-test; Significance was set at $p < 0.05$; [a] $p < 0.01$ compared to the Sham group; [b] $p < 0.001$ compared to the Sham group.

Figure S1. Spleens from the Sham and ACLT groups on day 30, 60 and 90 were removed. Serial sections were stained with antibodies against CD8$^+$ and TIMP-1. The section showed from upper and lower panels were from the same section. Only the Sham group on day 90 was shown. In the ACLT group mice, increased TIMP-1-expressing CD8$^+$ T cells were seen in spleens compared to those in the Sham group 90 days post-surgery (200× magnification; scale bar = 50 μm). The CD8- and TIMP-1-double positive cells were indicted with white insets. The TIMP-1 single positive cells were indicated with red insets. The highest number of double-positive cells was found in the spleens of mice 90 days after surgery. The number decreased progressively in those of mice on day 60, and day 30 at sacrifice.

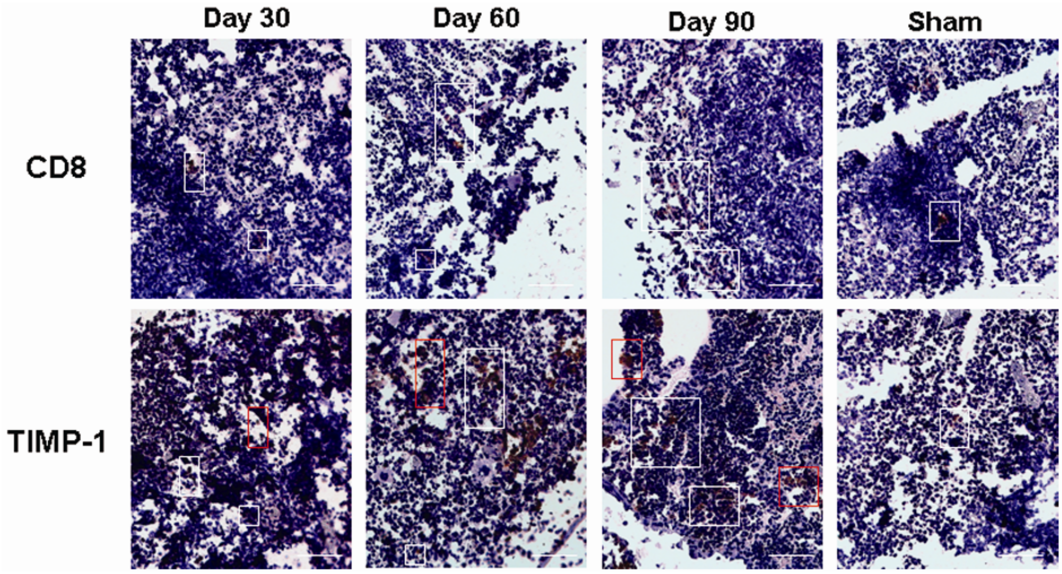

© 2013 by the authors; licensee MDPI, Basel, Switzerland. This article is an open access article distributed under the terms and conditions of the Creative Commons Attribution license (http://creativecommons.org/licenses/by/3.0/).
